# Comparison and Evaluation of GHG Emissions during Simulated Thermophilic Composting of Different Municipal and Agricultural Feedstocks

**DOI:** 10.3390/ijerph20043002

**Published:** 2023-02-09

**Authors:** Jianfei Zeng, Frederick C. Michel, Guangqun Huang

**Affiliations:** 1 Institute of Environment and Sustainable Development in Agriculture, Chinese Academy of Agricultural Sciences, Beijing 100081, China; 2 Department of Food, Agricultural and Biological Engineering, CFAES Wooster, The Ohio State University, Wooster, OH 44691, USA; 3 Engineering Laboratory for AgroBiomass Recycling & Valorizing, College of Engineering, China Agricultural University, Beijing 100083, China

**Keywords:** GHG emissions, thermophilic composting, dairy manure, chicken litter, biosolids, yard trimmings, food waste

## Abstract

Composting is widely used to recycle a variety of different organic wastes. In this study, dairy manure, chicken litter, biosolids, yard trimmings and food waste were selected as representative municipal and agricultural feedstocks and composted in simulated thermophilic composting reactors to compare and evaluate the GHG emissions. The results showed that the highest cumulative emissions of CO_2_, CH_4_ and N_2_O were observed during yard trimmings composting (659.14 g CO_2_ kg^−1^ DM), food waste composting (3308.85 mg CH_4_ kg^−1^ DM) and chicken litter composting (1203.92 mg N_2_O kg^−1^ DM), respectively. The majority of the carbon was lost in the form of CO_2_. The highest carbon loss by CO_2_ and CH_4_ emissions and the highest nitrogen loss by N_2_O emission occurred in dairy manure (41.41%), food waste (0.55%) and chicken litter composting (3.13%), respectively. The total GHG emission equivalent was highest during food waste composting (365.28 kg CO_2_-eq ton^−1^ DM) which generated the highest CH_4_ emission and second highest N_2_O emissions, followed by chicken litter composting (341.27 kg CO_2_-eq ton^−1^ DM), which had the highest N_2_O emissions. The results indicated that accounting for GHG emissions from composting processes when it is being considered as a sustainable waste management practice was of great importance.

## 1. Introduction

With increasing rates of organic solid waste production from different sources, the use of aerobic composting is growing as an effective and sustainable treatment process to stabilize, dry, reduce the volume of and recycle organic solid wastes and nutrients [1,2]. Carbon dioxide (CO_2_), methane (CH_4_) and nitrous oxide (N_2_O) are among the major pollutant gases produced during this process. CH_4_ and N_2_O are well-known greenhouse gases with global warming potentials 28.5 and 264.8 times higher than that of CO_2_, estimated over a 100-year period [3]. CH_4_ contributed 18% of the global net anthropogenic GHG emissions in 2019 [4] and N_2_O contributed 7%, largely from agricultural operations. N_2_O is also considered to be an important factor in ozone depletion [5].

The mechanism of CH_4_ emission from aerobic composting includes CH_4_ generation in the strictly anaerobic core of the composting matrix and CH_4_ oxidation in the aerobic layer on a particle scale [6,7,8]. The insufficient supply and/or inhomogeneous distribution of oxygen (O_2_) reduces the dissolved oxygen content at the liquid/gas interface of compost particles and further reduces the thickness of the aerobic layer, which enhances anaerobic decomposition. Production of N_2_O can occur during both well aerated and oxygen-limited composting through incomplete nitrification and denitrification processes [9,10]. During incomplete denitrification, nitrite is reduced to nitric oxide (NO) by nitrite reductase, which is further reduced to N_2_O by nitric oxide reductase [11]. Incomplete denitrification is promoted by limited oxygen and low pH conditions [12]. N_2_O production can also occur during incomplete nitrification, during which ammonia (NH_3_) is oxidized to hydroxylamine (NH_2_OH) by ammonia mono-oxygenase (AMO), subsequently oxidized to nitrite by hydroxylamine oxidoreductase and further oxidized to nitrate by nitrite oxidoreductase [13,14]. N_2_O is produced and released through incomplete oxidation of the NH_2_OH to nitrite or through chemical decomposition of intermediates when aeration is not limited [15].

There are many factors that influence GHG emissions during composting, which include waste type, management system [16,17,18,19], treatment duration [10], climatic condition [10,20] and experiment scale [21,22]. These were systematically reviewed by Pardo et al. (2015) [23]. Among these variables, the type of organic solid waste played an important role in the dynamics and cumulative emissions of GHGs. A series of studies have been conducted and focused on GHG emissions during dairy manure composting [19,24,25,26], chicken manure composting [27,28,29,30,31], biosolids composting [32,33,34,35], green waste composting [36,37,38] and food waste composting [10,38,39,40]. However, these studies adopted different aeration methods (continuous and intermittent aeration), aeration rates (high, moderate and low aeration rate), bulking agent types (crop straw, rice hull, sawdust, etc.) and bulking agent addition ratio (0~25%), leading to the data and results being of low comparability. There has been no systematic comparison and evaluation of GHG emissions during the composting of different types of organic solid waste under identical experimental conditions.

Therefore, the aim of this study was to investigate the dynamics and cumulative emissions of CO_2_, CH_4_ and N_2_O during the thermophilic composting of five different types of organic solid wastes (including dairy manure, chicken litter, biosolids, yard trimmings and food waste) amended with wheat straw under an identical experimental condition. Carbon and nitrogen losses and the total GHG emission equivalent from the different types of organic solid waste were also compared and evaluated.

## 2. Materials and Methods

### 2.1. Sources and Types of Organic Solid Wastes

Dairy manure, fresh grass, leaves and wheat straw were obtained from the OSU CFAES Wooster campus in Wooster, OH, USA. Chicken litter was obtained from Rock creek farm (Jeromesville, OH, USA). Biosolids also known as sewage sludge were collected from a mesophilic liquid anaerobic digester operated by KB BioEnergy, Inc. (Akron, OH, USA). Food waste was collected from Paradise Composting in Wooster, OH, USA. Yard trimmings consisted of fresh grass (90% *w*/*w*) and leaves (10% *w*/*w*) that were chopped into about 1 cm lengths and mixed together. Chicken litter, which had a low moisture content of 25.47%, was firstly amended with deionized water to achieve an appropriate moisture content (67.70%). Wheat straw was chopped into 3−5 cm lengths and used as a bulking agent. Different types of waste and wheat straw were mixed at a ratio of 12:1 on a wet weight basis. The physicochemical properties of the raw materials and initial mixtures are shown in Table 1.

### 2.2. Reactor System

**Figure 1:**
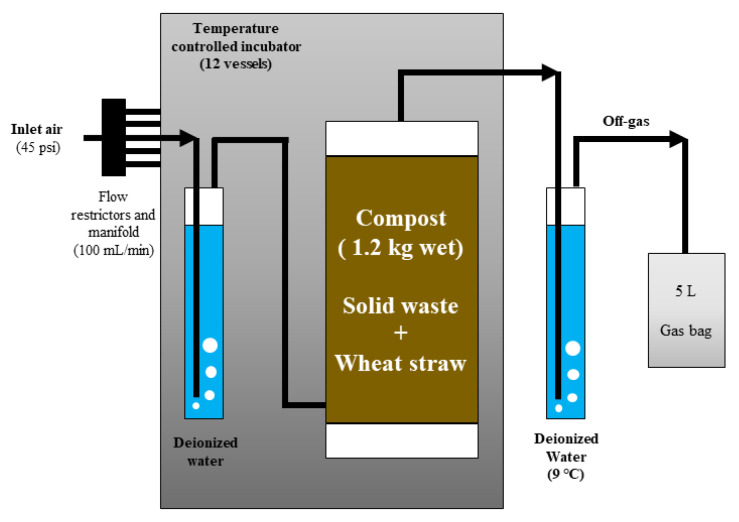
Schematic of the laboratory scale composting system.

The experiment was conducted using a bench scale composting system (Figure 1) described by Lin et al. (2014) [37] and Grewal et al. (2006) [41] to simulate thermophilic windrow composting conditions. For each treatment, 1.2 kg of mixture was loaded into 4-L compost reactors and duplicate reactors were tested for each treatment (*n* = 2). Each reactor was continuously aerated at a rate of 100 mL/min according to Lin et al. (2014) [37] and Grewal et al. (2006) [41] to simulate passive convection. Yard trimmings were an exception since the 4-L compost reactors could only be filled with 0.6 kg of Y_T because of the low bulk density of YT. The reactors were placed into an incubator with a set point temperature of 55 °C to simulate the thermophilic phase of composting. The mixtures were composted for 45 days. Off-gas from each reactor passed through a water bath at 9 °C to condense moisture and the de-watered off-gas was then collected in a gas sampling bag (Restek Corporation, 22953, Bellefonte, PA, USA).

### 2.3. Analytical Methods

Compost samples were collected at the beginning and the end of the 45-day incubation. Measurements of organic matter (OM), dry matter (DM) and moisture content (MC) were conducted according to standard methods [42]. Total nitrogen (TN) and total carbon (TC) were measured using an elemental analyzer (Elementar Americas, Elementar Vario Max CNS, Mt. Laurel, NJ, USA) and C/N was calculated as the ratio of TC to TN. Air filled porosity (AFP) and bulk density (BD) were measured based on the quick method [42,43,44].

Gas samples were collected every two days over the first 8 days, every five days over the next 25 days and every six days thereafter. The concentrations of CO_2_, CH_4_ and N_2_O were measured using an integrated gas analyzer (Gasera Ltd. Gasera One, Turku, Finland) equipped with different sensors. The gas emission rates (*E_aer_*) were calculated according to Zeng et al. (2018) [45] as follows:

$$E_{aer}=\frac{P\cdot Q\cdot M\cdot \left(C_{out}-C_{in}\right)}{R\cdot T\cdot m_{1,DM}}\times 60\times 24\times 1000$$

where *E_aer_* is the emission rate of CO_2_, CH_4_ or N_2_O during aeration (mg kg^−1^ DM day^−1^), *P* is the atmospheric pressure at elevation of measurement (atm), *Q* is the aeration rate (0.1 L min^−1^), *M* is the molecular weight of different gases (g mol^−1^), *Cout* is the gas concentration in the outlet airflow during aeration (ppmv), *C_in_* is the gas concentration in the inlet airflow during aeration (ppmv), *R* is the ideal gas constant (0.08206 L atm mol^−1^ K^−1^), *T* is the temperature of inlet airflow (K), $m_{1,DM}$ is the weight of dry matter in the initial mixture (kg), 60 is the conversion factor from min to h, 24 is the conversion factor from h to day, and 1000 is the conversion factor from g to mg.

Cumulative emissions were calculated by summing the daily emissions [46]. Data for non-measured days were obtained by averaging the data from closest measured days or using duplicate reactor data [9].

### 2.4. Statistical Analysis

Data are expressed as the means ± standard deviations of the duplicate measurements. All figures were drawn using the software program OriginPro 9.1 (OriginLab Corp., Northampton, MA, USA).

## 3. Results and Discussion

### 3.1. CO_2_ Emissions

The five different composts had a range of different initial C/N and moisture contents. The C/N ranged widely from 10.86 for the sewage sludge (S_S) to 24.18 for food waste (F_W) compost. The moisture contents of the mixtures were more similar ranging from 65.92% for the chicken litter compost (C_L) to 77.84% for the dairy manure compost (Table 1).

**Figure 2:**
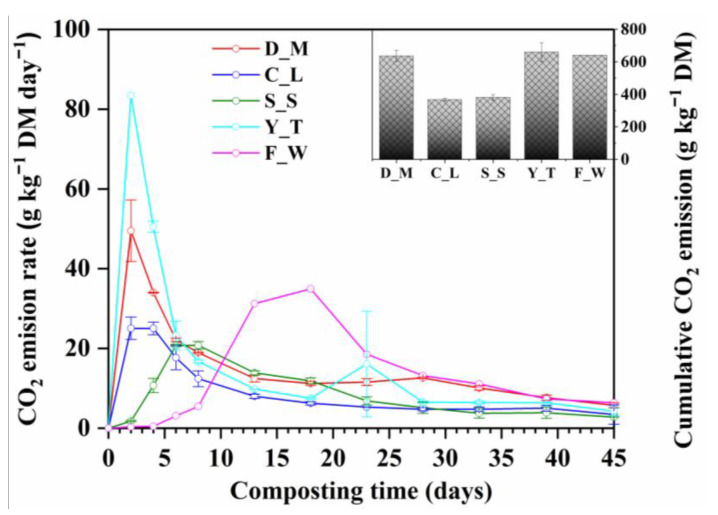
CO_2_ emission rate and cumulative CO_2_ emission during composting of different types of organic solid waste. D_M: dairy manure + wheat straw; C_L: chicken litter + wheat straw; S_S: biosolids + wheat straw, Y_T: yard trimming + wheat straw; F_W: food waste + wheat straw.

**Figure 3:**
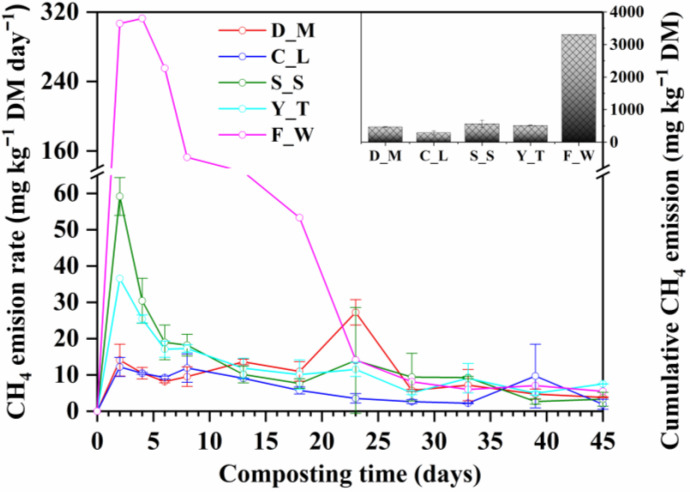
CH_4_ emission rate and cumulative CH_4_ emission during composting of different types of organic solid waste. D_M: dairy manure + wheat straw; C_L: chicken litter + wheat straw; S_S: biosolids + wheat straw, Y_T: yard trimming + wheat straw; F_W: food waste + wheat straw.

The CO_2_ emission rates during composting rapidly increased and peaked on Day 2 in D_M (49.51 g kg^−1^ DM d^−1^), C_L (25.05 g kg^−1^ DM d^−1^) and Y_T (83.44 g kg^−1^ DM d^−1^) treatments (Figure 2). They then markedly decreased during the next 6 days, a trend which is commonly observed [32,47] owing to the fast decomposition of easily degradable compounds [48]. The CO_2_ emission rates from S_S and F_W treatments peaked later on Days 8 and 18, respectively. This was likely due to the lower porosity and air permeability of the de-watered sewage sludge particles [49], and food waste mixture was the lowest in air-filled porosity (Table 1), leading to anaerobic reactions initially being dominant in S_S and F_W treatments. This result coincided with high CH_4_ emission rates from the S_S and F_W treatments (Figure 3). The Y_T treatment had the highest maximum CO_2_ emission rate (83.44 g kg^−1^ DM d^−1^) due to the high air-filled porosity and low bulk density of the yard trimmings mixture (Table 1), leading to strong aerobic microbial activity and rapid degradation [28]. In contrast, the S_S treatment had a lowest maximum CO_2_ emission rate of 20.77 g kg^−1^ DM d^−1^ due to the low porosity and air permeability. The C_L treatment was also found to have a relatively low CO_2_ emission rate (25.05 g kg^−1^ DM d^−1^) similar to the S_S treatment. The reason for this might be that the chicken litter was partially stabilized in the henhouse before being collected.

After 45-days of composting, the Y_T treatment generated the most CO_2_ (659.14 g kg^−1^ DM), followed by the F_W (640.94 g kg^−1^ DM), D_M (635.87 g kg^−1^ DM), S_S (380.32 g kg^−1^ DM) and C_L (367.15 g kg^−1^ DM) treatments, respectively.

### 3.2. CH_4_ Emissions

The CH_4_ emission rates during composting peaked on Day 2 or 3 (Figure 3), which was much faster than the results observed in previous studies [10,18,27]. This may be due to the higher temperature of 55 ± 0.3 °C used for the treatments in this study, which may have enhanced the activities of methanogens and promoted the oxygen depletion [7,10].

The highest CH_4_ emission rate was observed in the F_W treatment (312.75 mg kg^−1^ DM d^−1^), and this was much greater than those observed in the S_S (59.16 mg kg^−1^ DM d^−1^), Y_T (36.4 mg kg^−1^ DM d^−1^), D_M (14.08 mg kg^−1^ DM d^−1^) and C_L (12.20 mg kg^−1^ DM d^−1^) treatments. Likewise, the highest cumulative CH_4_ emission was also observed from the F_W treatment (3308.85 mg kg^−1^ DM) and this was more than 5.8 times greater than methane emissions from the S_S (563.86 mg kg^−1^ DM), Y_T (513.57 mg kg^−1^ DM), D_M (466.21 mg kg^−1^ DM) and C_L (286.71 mg kg^−1^ DM) treatments (Figure 3). Both the emission rate and the cumulative emissions of CH_4_ in the F_W treatment were much higher than those reported by Xu et al. (2021), which were about 38 mg kg^−1^ DM d^−1^ and 490 mg kg^−1^ DM, respectively [38]. Considering that the CO_2_ emission from the Food Waste treatment was also high among the five treatments (Table 2), the O_2_ supply in F_W treatment was thought to be sufficient as a whole. So, the high remission of CH_4_ from the Food Waste treatment may have been due to the short circuit of airflow in the reactor and anaerobic reaction in the partial zone caused by the lowest air filled porosity and second highest bulk density (Table 1) and more labile organic matter in the F_W treatment; otherwise, the emission of CH_4_ from Xu et al. (2021) was low because the amendment of food waste with garden wastes could effectively reduce CH_4_ emission [38]. The CH_4_ emission from S_S was also obviously higher than that from D_M and C_L treatments even though the air-filled porosity of S_S was higher and bulk density of S_S was lower, likely because sewage sludge was compacted and of very low bulk density after being de-watered [35], which led to a lower air permeability and more intensive anaerobic reaction [49] The results illustrate that pre-treatment and amendment of food waste and sewage sludge with bulking agents is very necessary and important to improve air permeability and reduce CH_4_ emissions.

### 3.3. N_2_O Emission

**Figure 4:**
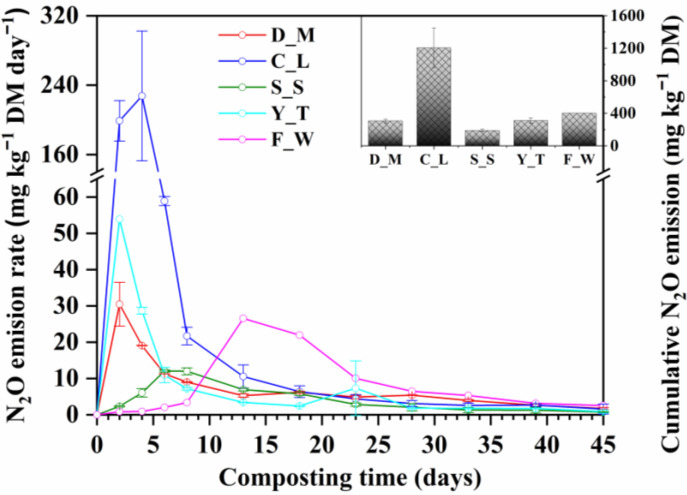
N_2_O emission rate and cumulative N_2_O emission during composting of different types of organic solid waste. D_M: dairy manure + wheat straw; C_L: chicken litter + wheat straw; S_S: biosolids + wheat straw, Y_T: yard trimming + wheat straw; F_W: food waste + wheat straw.

Significant N_2_O emissions were immediately observed from the C_L, Y_T and D_M treatments (Figure 4). These emission patterns were similar to those observed in the literature for similar composts [17,38,48]. This has two different explanations: one is that the presence of NO_3_^−^ in the organic solid wastes led to high N_2_O emission by denitrification [48], while the other is that nitrification of the NH_4_^+^ in the initial mixtures caused high N_2_O emissions [35,50,51]. The N_2_O emission rates in the S_S and F_W treatments were relatively low during the initial period and did not peak until Days 6 and 13, respectively, which was consistent with rates observed by Yuan et al. (2016) [35] and He et al. (2000) [40] for these types of composts. The reason for the lower rates may be the inhibition of nitrification by the thermophilic composting temperature. The N_2_O emission rate of the C_L treatment (227.56 mg kg^−1^ DM d^−1^) was much higher than that from other treatments because the chicken litter (a mixture of chicken manure, bedding material, waste feed and feathers) likely contained more easily degradable nitrogen compounds. The production and emission of N_2_O from the C_L, Y_T and D_M treatments concentrated within the first 8 days, and this accounted for 82.44%, 62.23% and 42.82% of the total N_2_O emissions, respectively. The total N_2_O emissions were greatest from the C_L (1203.92 mg kg^−1^ DM), followed by the F_W (400.11 mg kg^−1^ DM), Y_T (310.14 mg kg^−1^ DM) and D_M (305.15 mg kg^−1^ DM) and lowest from the S_S treatment (246.05 mg kg^−1^ DM). Compared with the total N_2_O emissions from the C_L treatment, Wu et al. (2021) reported a much lower N_2_O emission during the electric field-assisted aerobic composting of chicken manure mainly because alternating magnetic field could weaken the expressions of the *amo*A, *nar*G and *nir*S functional genes but enhance the expression of the *nos*Z functional gene and mitigate N_2_O [30].

### 3.4. Carbon and Nitrogen Losses

**Figure 5:**
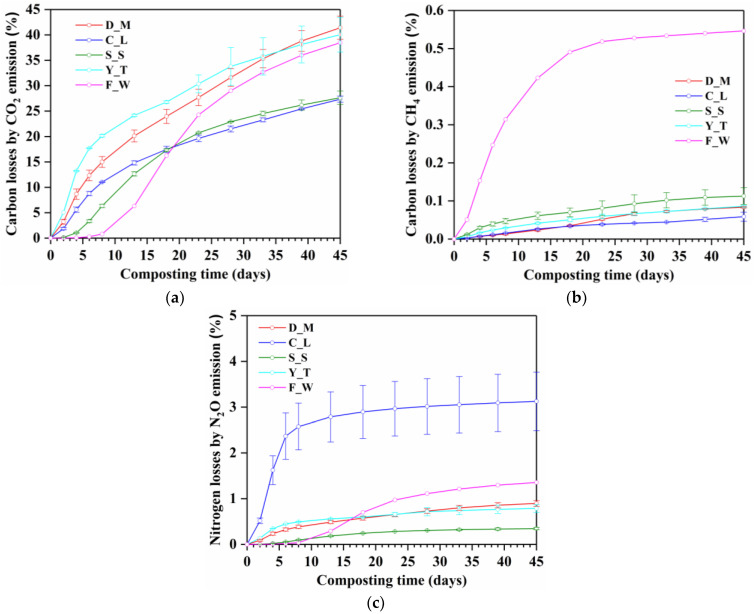
Carbon losses by CO_2_ emission (**a**), CH_4_ emission (**b**) and nitrogen loss by N_2_O emission (**c**) during composting of different types of organic solid waste. D_M: dairy manure + wheat straw; C_L: chicken litter + wheat straw; S_S: biosolids + wheat straw, Y_T: yard trimming + wheat straw; F_W: food waste + wheat straw.

CO_2_ emission rate is an indicator of the overall microbial activity and can reflect the degree of aerobic degradation. As presented in Figure 5, about 27.34 to 41.41% of the total carbon was lost as CO_2_, which accounted for more than 98.60% of the total carbon losses by CO_2_ and CH_4_ emissions, indicating that the majority of the initial carbon lost during composting from all treatments was in the form of CO_2_. Lin et al. (2014) found that the total carbon loss by CO_2_ accounted for less than 50% in solid-state anaerobic digestion and almost 100% in composting of yard trimmings [37]. Yuan et al. (2016) also reported that more than 98% of the total carbon was lost in the form of CO_2_ [35]. This result proved that the O_2_ supply in all treatment was thought to be sufficient as a whole even though the F_W treatment emitted large amount of CH_4_. The CO_2_ emissions from the D_M and Y_T were higher than those from the other treatments because the initial dairy manure and yard trimming mixtures were of higher organic matter contents.

The amount of carbon lost as CH_4_ was low ranged from 0.06% to 0.55% of the total carbon, among which the highest carbon loss by CH_4_ emission occurred in F_W treatment (Figure 5b) because of the lowest air-filled porosity and second highest bulk density of the food waste mixture.

Less nitrogen was lost as N_2_O (0.35 to 3.13% of the total nitrogen) because the majority of the initial nitrogen lost during composting was in the form of NH_3_ [9]. The highest nitrogen loss by N_2_O emission was found from C_L due to the higher N content and more easily degradable nitrogen compounds in the chicken litter.

### 3.5. GHG Emission Equivalent

The gas with the greatest contribution to GHG emission equivalents for all treatments was CO_2_ (Table 2) and this was as high as 5 times greater than the GHG emission equivalent contributions by N_2_O and CH_4_ in the D_M and Y_T treatments. The total GHG emission equivalent (excluding CO_2_) was highest from F_W (365.28 kg CO_2_-eq ton^−1^ DM) mainly due to the highest CH_4_ emissions (259.33 kg CO_2_-eq ton^−1^ DM) from the F_W. The C_L treatment (341.27 kg CO_2_-eq ton^−1^ DM) presented the second highest total GHG emission equivalent (excluding CO_2_) because of the highest N_2_O emissions (318.80 kg CO_2_-eq ton^−1^ DM) from C_L. The S_S treatment produced the lowest total GHG emission equivalent (94.08 kg CO_2_-eq ton^−1^ DM) owing to the lowest N_2_O emissions (49.89 kg CO_2_-eq ton^−1^ DM). González et al. (2020) controlled the aeration flow rate by the oxygen uptake rate (OUR) during sewage sludge composting and this mode could effectively reduce CH_4_ emission but emitted much more N_2_O compared with the present study [34].

The whole composting process was divided into two stages: days 0~13 were the degradation stage and days 13~45 were the maturation stage. For each, the CO_2_, CH_4_, N_2_O and total GHG emissions from the C_L, S_S and Y_T treatments were concentrated within the degradation stage, which accounted for more than 44.74% of the total emissions for each, and a similar trend was very common in the literature [29,32,37]. More than 46.43% of the CO_2_, N_2_O and total GHG emissions from the D_M treatments was also concentrated within the degradation stage, but 71.50% of the CH_4_ was emitted during the maturation stage because of the sudden increase on Day 23. For F_W treatments, however, 77.42% of the CH_4_ emission occurred during the degradation stage, but 83.63% of the CO_2_ emission occurred after the degradation stage; this result indicated that the anaerobic reaction was initially dominant in the degradation stage and the aerobic reaction gradually recovered afterwards with the consumption of organic matter and improvement of air-filled porosity and air permeability. Otherwise, 78.59% of the N_2_O emission occurred during, demonstrating that N_2_O from F_W treatments was mainly produced and released through incomplete nitrification in the maturation stage, during which ammonia (NH_3_) is oxidized to hydroxylamine (NH_2_OH) by ammonia mono-oxygenase (AMO), subsequently oxidized to nitrite by hydroxylamine oxidoreductase and further oxidized to nitrate by nitrite oxidoreductase [13,14].

## 4. Conclusions

Although usually thought of as an aerobic process emitting only CO_2_, significant amounts of other greenhouse gases can be formed and emitted during the thermophilic composting of municipal and agricultural feedstocks as shown by this and other studies. However, overall, the majority of the initial carbon lost during composting from all treatments was in the form of CO_2_. The highest carbon loss by CO_2_ emissions occurred during dairy composting and the highest CO_2_ emission rates were observed from yard trimmings, dairy manure and food waste composts, in that order. Food waste compost emitted the greatest amounts of CH_4_ and chicken litter compost emitted the greatest amounts of N_2_O among the five different feedstocks. Non-CO_2_ GHG emissions from these two feedstocks were nearly three times greater than those from yard trimmings, dairy manure and biosolids composts. The overall total GHG emission equivalent emissions were also greatest during food waste composting, as a result of both high CH_4_ and N_2_O emissions, followed by chicken litter compost which had the highest cumulative N_2_O emissions. These results of this study may not represent all of the processes that occur in full scale windrows which have wider ranges of temperatures, moisture contents and interstitial oxygen concentrations than those used in the simulation reactors in this study. However, they point to the importance of accounting for GHG emissions from composting processes when it is being considered as a sustainable waste management practice. Pre-treatment and amendment of municipal and agricultural waste with bulking agents is necessary and important to improve air permeability and reduce CHG emissions.

## Figures and Tables

**Table 1:** Basic physicochemical properties of the raw materials and initial mixtures.

Materials ^a^		MC (%) ^b^	OM (%) ^c^	TC (%) ^c^	TN (%) ^c^	C/N ^c^	AFP (%) ^b^	BD (kg/m^3^) ^b^
Raw materials	Dairy manure	84.94 ± 0.15	87.15 ± 0.78	43.55 ± 0.44	2.58 ± 0.06	16.86 ± 0.19	-	-
	Chicken litter	25.47 ± 0.09	80.25 ± 0.07	37.34 ± 2.56	3.22 ± 0.20	11.61 ± 0.09	-	-
	Biosolids	70.22 ± 0.19	67.91 ± 0.82	38.19 ± 0.00	4.03 ± 0.02	9.47 ± 0.03	-	-
	Grass	70.68 ± 0.68	89.60 ± 0.13	41.85 ± 0.06	2.90 ± 0.00	14.45 ± 0.01	-	-
	Leaf	27.65 ± 0.20	95.26 ± 0.24	47.62 ± 0.04	0.96 ± 0.00	49.48 ± 0.06	-	-
	Food waste	72.48 ± 1.27	80.60 ± 2.75	47.02 ± 1.05	4.76 ± 0.12	9.88 ± 0.04	-	-
	Wheat straw	8.95 ± 0.10	94.33 ± 0.23	43.17 ± 0.40	0.49 ± 0.01	88.20 ± 1.96	-	-
Mixtures	D_M	77.84 ± 0.77	90.07 ± 0.15	41.88 ± 0.15	2.17 ± 0.01	19.31 ± 0.13	66.50 ± 0.09	363.83 ± 14.53
	C_L	65.92 ± 0.50	83.67 ± 0.12	36.62 ± 0.14	2.45 ± 0.03	14.94 ± 0.12	68.28 ± 0.17	285.07 ± 3.77
	S_S	68.06 ± 1.13	72.87 ± 0.89	37.52 ± 0.42	3.46 ± 0.01	10.86 ± 0.12	73.54 ± 3.30	242.67 ± 21.40
	Y_T	70.06 ± 0.48	92.09 ± 0.52	44.88 ± 2.51	2.51 ± 0.00	17.89 ± 1.02	85.44 ± 2.84	88.92 ± 2.24
	F_W	67.14 ± 1.42	84.00 ± 1.11	45.43 ± 0.06	1.88 ± 0.04	24.18 ± 0.43	65.85 ± 2.63	345.70 ± 3.54

^a^ Moisture content (MC), organic matter content (OM), total carbon content (TC), total nitrogen content (TN), ratio of TC to TN (C/N), air filled porosity (AFP), bulk density (BD), dairy manure + wheat straw (D_M), chicken litter + wheat straw (C_L), biosolids + wheat straw (S_S), yard trimming + wheat straw (Y_T) and food waste + wheat straw (F_W). ^b^ Measurement based on wet weight. ^c^ Measurement based on dry weight.

**Table 2:** GHG emission equivalent.

Gases	Time (Days)	GHG Emission Equivalent (kg CO_2_-eq ton^−1^ DM) ^a^				
		D_M ^b^	C_L ^b^	S_S ^b^	Y_T ^b^	F_W ^b^
CO_2_	0~13	308.75 ± 18.06	199.00 ± 5.68	173.92 ± 5.91	397.49 ± 3.27	104.95
	13~45	327.12 ± 17.10	168.15 ± 13.85	206.40 ± 12.19	261.65 ± 59.65	535.99
	sum	635.87 ± 35.16	367.15 ± 8.17	380.32 ± 18.10	659.14 ± 56.37	640.94
CH_4_	0~13	10.41 ± 1.48	10.05 ± 0.53	24.00 ± 3.80	19.46 ± 1.01	200.77
	13~45	26.13 ± 2.24	12.42 ± 4.02	20.19 ± 4.78	20.76 ± 0.02	58.56
	sum	36.54 ± 0.76	22.47 ± 4.55	44.19 ± 8.58	40.25 ± 1.03	259.33
N_2_O	0~13	44.07 ± 3.43	284.17 ± 55.68	26.53 ± 1.54	58.45 ± 0.29	22.68
	13~45	36.73 ± 2.29	34.63 ± 9.48	23.36 ± 2.33	23.68 ± 9.35	83.27
	sum	80.80 ± 5.72	318.80 ± 65.15	49.89 ± 3.87	82.12 ± 9.06	105.95
Total GHG	0~13	54.49 ± 4.91	294.22 ± 56.21	50.53 ± 5.34	77.91 ± 1.30	223.45
	13~45	62.86± 0.04	47.05 ± 13.49	43.56 ± 7.11	44.47 ± 9.33	141.83
	sum	117.34 ± 4.95	341.27 ± 69.71	94.08 ± 12.45	122.38 ± 8.03	365.28

^a^ Global warming potential calculation: 1 mol CH_4_ = 28.5 mol CO_2_-eq, 1 mol N_2_O = 264.8 mol CO_2_-eq [3]. ^b^ Dairy manure + wheat straw (D_M), chicken litter + wheat straw (C_L), biosolids + wheat straw (S_S), yard trimming + wheat straw (Y_T) and food waste + wheat straw (F_W).

## Data Availability

Data used in this study are not available upon appropriate requests to the corresponding author.
